# Author Correction: A comprehensive model for assessment of liver stage therapies targeting *Plasmodium vivax* and *Plasmodium falciparum*

**DOI:** 10.1038/s41467-018-04817-1

**Published:** 2018-06-08

**Authors:** Alison Roth, Steven P. Maher, Amy J. Conway, Ratawan Ubalee, Victor Chaumeau, Chiara Andolina, Stephen A. Kaba, Amélie Vantaux, Malina A. Bakowski, Richard Thomson-Luque, Swamy Rakesh Adapa, Naresh Singh, Samantha J. Barnes, Caitlin A. Cooper, Mélanie Rouillier, Case W. McNamara, Sebastian A. Mikolajczak, Noah Sather, Benoît Witkowski, Brice Campo, Stefan H. I. Kappe, David E. Lanar, François Nosten, Silas Davidson, Rays H. Y. Jiang, Dennis E. Kyle, John H. Adams

**Affiliations:** 1 Department of Global Health, College of Public Health, Center for Global Health and Infectious Diseases Research, University of South Florida, 3720 Spectrum Blvd 404, 33612 Tampa, FL USA; 2 Center for Tropical and Emerging Global Diseases, University of Georgia, 500 DW Brooks Dr. Suite 370, 30602 Athens, GA USA; 3 Department of Entomology, Armed Forces Research Institute of Medical Sciences (AFRIMS), 315/6 Rajvithi Rd, 10400 Bangkok, Thailand; 4 Centre for Tropical Medicine and Global Health, Nuffield Department of Medicine, University of Oxford, Oxford, UK; 5 Shoklo Malaria Research Unit, Mahidol Oxford Research Unit, Faculty of Tropical Medicine, Mahidol University, 68/30 Bantung Rd, 63110 Mae Sot, Tak Thailand; 6 Malaria Vaccine Branch, Walter Reed Army Institute of Research, 503 Robert Grant Ave, 20910 Silver Spring, MD USA; 7 Malaria Molecular Epidemiology Unit, Institut Pasteur du Cambodge, 5 Boulevard Monivong-PO Box 983, 12 201 Phnom Penh, Cambodia; 8 California Institute for Biomedical Research (Calibr), 11119N. Torrey Pines Rd, Suite 100, 92037 La Jolla, CA USA; 9 Medicines for Malaria Venture, Pré-Bois Rd 20, 1215 Meyrin, Switzerland; 10 Center for Infectious Disease Research, 307 Westlake Ave N Suite 500, 98109 Seattle, WA USA

Correction to: *Nature Communications* 10.1038/s41467-018-04221-9, published online 09 May 2018.

The original version of this article contained an error in the spelling of Richard Thomson-Luque, which was incorrectly given as Richard Thomson Luque. This error has now been corrected in both the PDF and HTML versions of the Article.

