# Supplementary material for: A comprehensive model for assessment of liver stage therapies targeting Plasmodium vivax and Plasmodium falciparum
Source: Nat Commun. 2018 May 9;9:1837. doi: 10.1038/s41467-018-04221-9 (PMC5943321; doi:10.1038/s41467-018-04221-9)
Supplement: Supplementary file 1 — Supplementary Information [file 41467_2018_4221_MOESM1_ESM.pdf]

Supplementary Information for:

A comprehensive model for assessment of liver stage therapies targeting *Plasmodium vivax* and *Plasmodium falciparum*

Roth et al.

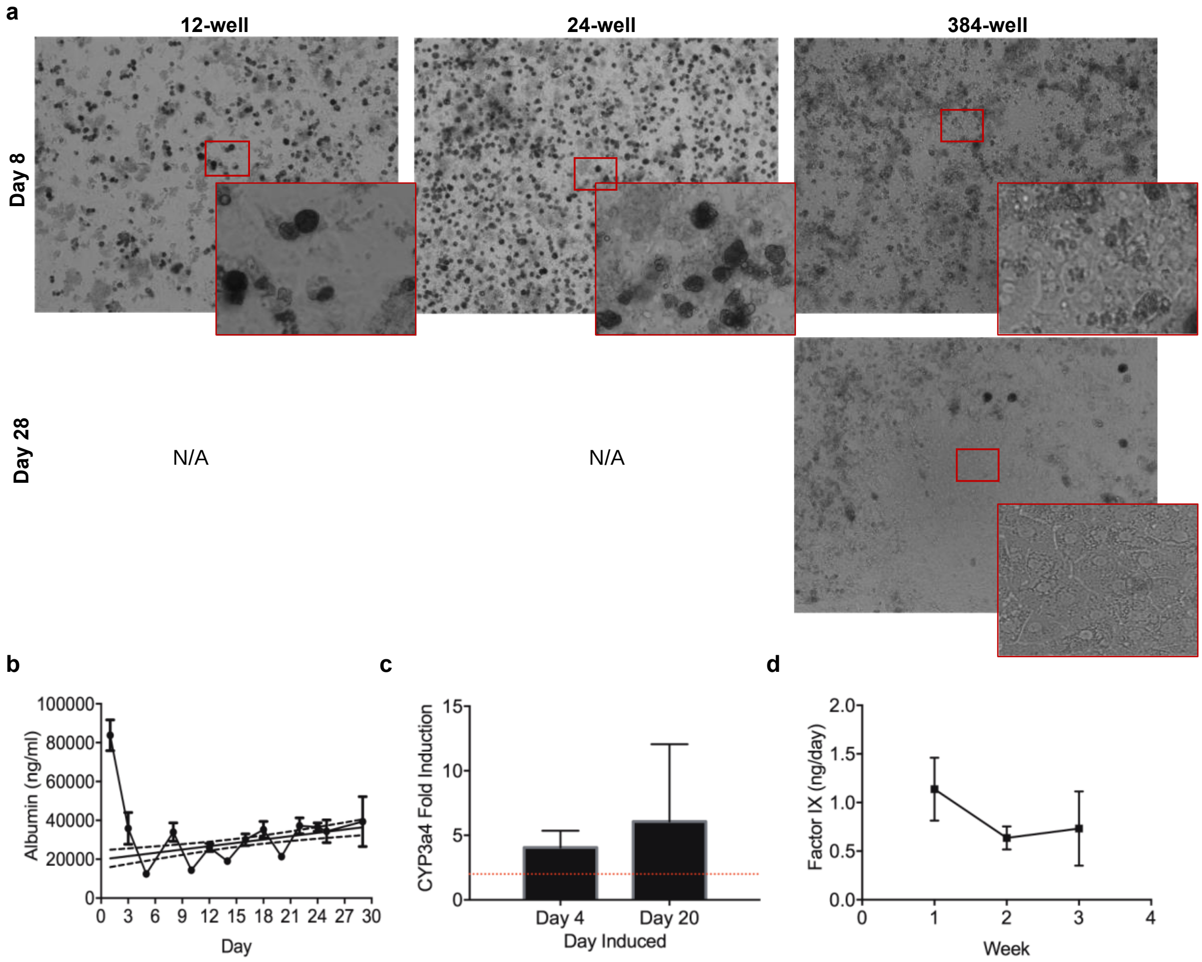

Supplementary Figure 1 | Functional assessment of primary human hepatocytes (PHHs) in commercial 384-well plate. (**a**) Long-term assessment of PHHs from Bioreclamation IVT, Inc. in 12-well, 24-well, and 384-well formats. Images taken in bright field at 20x, 0.4 NA showing days 8 and 28 post seed. At day 8, PHH in 12-well have detached while PHH in 24-well have begun apoptotic degradation and detachment. Only PHHs in 384-well have a persistent monolayer with polarized cells and visible tight junctions still present at day 28. Standard seed densities were used with 1×10^6 for 12-wells, 2.5×10^5 for 24-wells and 1.8×10^4 for 384-wells. (**b**) PPHs maintain stable albumin production for 30 days serving as a biomarker of normal liver function with active small molecule transports. **(c, d)** Similarly, induction of CYP3A4 shows active metabolism for at least 20 days (red line indicates minimum production level) accompanied with stable factor IX secretion for 3 weeks. Graph bars represent means with s.d. of experimental replicates (n = 6 (**b**), n = 4 (**c**, **d**)).

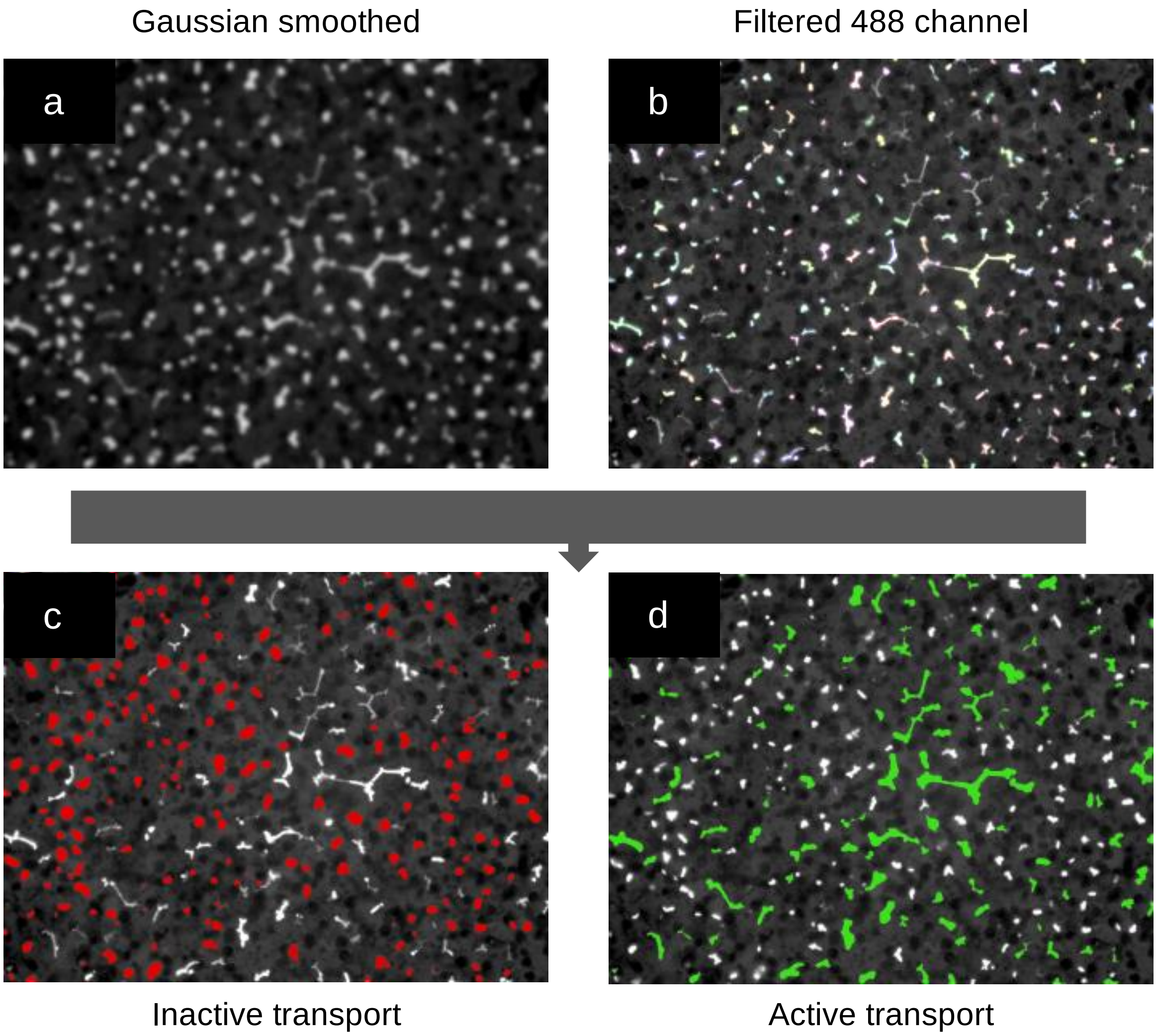

Supplementary Figure 2 | Post image analysis sequence of hepatobiliary formation. Primary human hepatocytes (PHHs) were stained live with CellTracker^{TM} Green CMFDA and images collected measuring multidrug resistant-associated protein 2 (MRP2) expression by accumulation of glutathione methylfluorescein within bile canaliculi. Monolayers of PHHs in microtiter wells were imaged in triplicates at pre-defined time points with z-plane stacks at every 2 µm (from -2–8 µm). Images were processed by first generating a maximum intensity projection image of the z-stack (6 images) and then generating a Gaussian image filter to enrich contrast of active bile canaliculi and normalize background intensity. Simultaneously, the image was filtered selecting for intensity of the FITC channel allowing for identification of hepatocyte cells incapable of transporting dye. An image calculation was applied (Gaussian filtered image **(a)** plus filtered FITC channel **(b)** and thresholds were set based on sum intensity, area, and roundness to classify all bile canaliculi as either inactive **(c)** or active transport **(d)**.

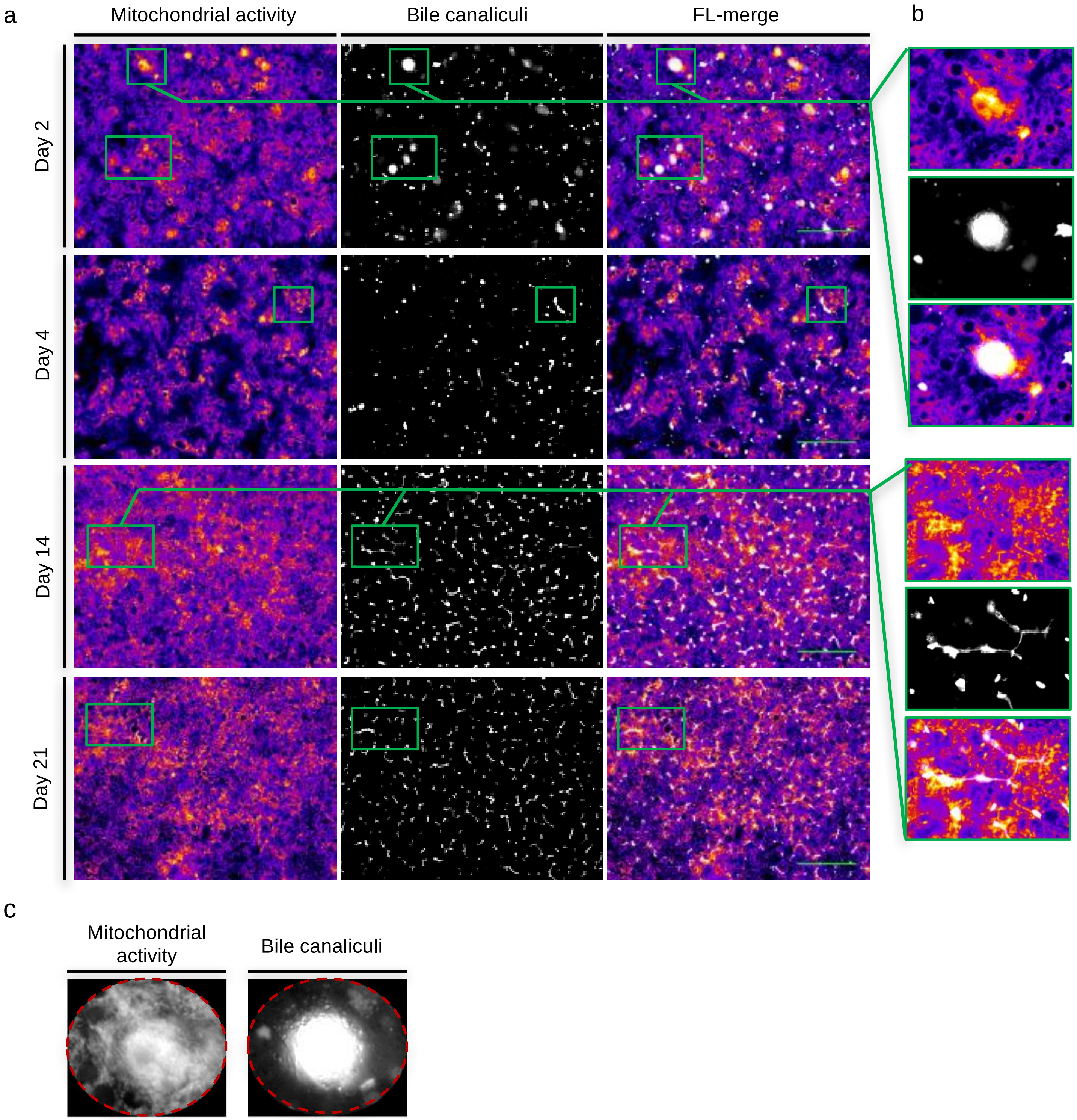

a
Mitochondrial activity
Bile canaliculi
FL-merge
b
Day 2
Day 4
Day 14
Day 21
c
Mitochondrial activity
Bile canaliculi

Supplemental Figure 3 | Association of bile canaliculi formation and growth with hepatocytes expressing high mitochondrial activity. (a) Collected images from days 2–21 were subjected to a post-analysis where z-stacked images were flattened as maximum projections and mitochondrial activity was colored by intensity level (purple = low, red = medium, yellow/orange = high). Additionally, bile canaliculi were transformed to grey scale and was merged with mitochondrial activity. **(b)** Re-mapping revealed a strong correlation of bile canaliculi formation and growth in the proximity of high fluorescence intensity of active mitochondria. (**c**) Representation of selected regions of interest (red dashed circle) from day 2 (**b**) for co-localization identification using Costes method automated threshold determination (M1, M2 = 1, tM1 = 0.657, tM2 = 0.739, Pearson's r value = 0.69). Scale bar, green, represents 100 µm.

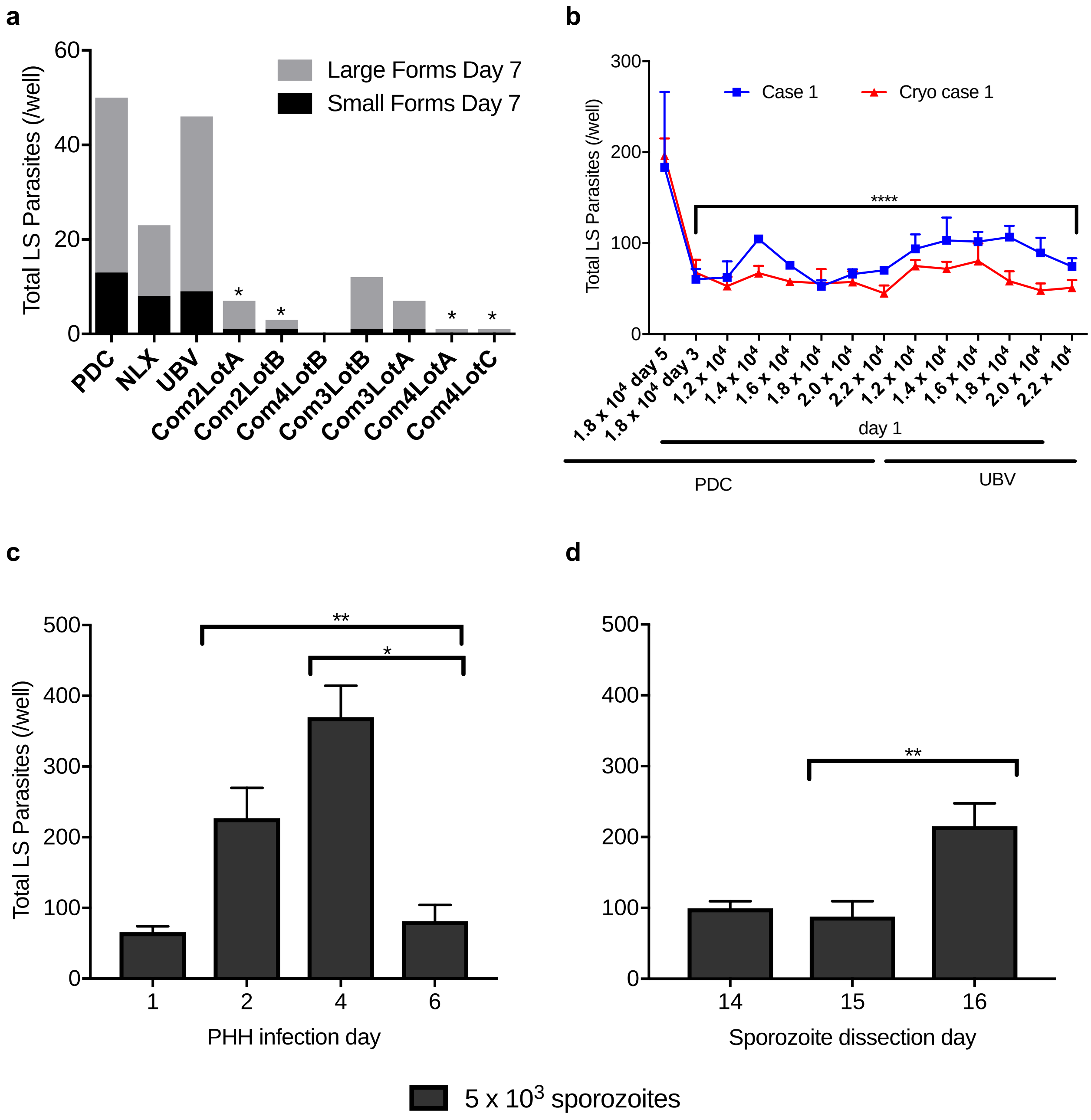
a
Total LS Parasites (/well)
Large Forms Day 7
Small Forms Day 7
PDC
NLX
UBV
Com2LotA
Com2LotB
Com4LotB
Com3LotB
Com3LotA
Com4LotA
Com4LotC
b
Total LS Parasites (/well)
Case 1
Cryo case 1
****
1.8 x 10⁴ day 5
1.8 x 10⁴ day 3
1.2 x 10⁴
1.4 x 10⁴
1.6 x 10⁴
1.8 x 10⁴
2.0 x 10⁴
2.2 x 10⁴
day 1
PDC
UBV
c
Total LS Parasites (/well)
PHH infection day
d
Sporozoite dissection day
5 x 10³ sporozoites

Supplemental Figure 4 | *P. vivax* susceptibility and phenotypic characterization in primary human hepatocytes (PHHs). (a) PHH donor lots (PDC, NLX, and UBV) from Bioreclamation IVT (BIVT) along with other commercially available lots were screened using previously cryopreserved *P. vivax* sporozoites showing significant reduction in sporozoite invasion and LS development in non-BIVT lots. **(b)** Seeding densities ranging from 1.2×10^4 to 2.2×10^4 were tested using two top tier PHH donor lots indicating the seeding density of 1.8×10^4 and infection of the PHH between day 4–5 post seed yield highest *P. vivax* LS parasites from separate cases. Fresh *P. vivax* sporozoites were inoculated at 5,000 per well while cryopreserved *P. vivax* sporozoites were inoculated at 20,000 per well. **(c)** *P. vivax* sporozoites harvested on day 16 were inoculated at 5,000 sporozoites per well into PDC PHHs at different days post seed, showing highest total LS parasites per well on day 4. **(d)** *P. vivax* sporozoites were harvested from mosquitoes day 14–16 post ingestion of infected blood meal and inoculated at 5,000 sporozoites per well into day 2 post-seeded PHH. Graph bars represent means with s.d. from experimental replicates (n = 2). Statistical significance determined using two-way ANOVA followed by Dunnett's multiple comparisons to PDC (**a**) or 1.8×10^4 at day 5 (**b**) where significance is presented by $P < 0.05$ (*) or $P < 0.0001$ (****). Statistical significance determined using one-way ANOVA (nonparametric) followed by Dunn's multiple comparison (**c, d**) where significance is presented by $P < 0.01$ (*) and $P < 0.005$ (**).

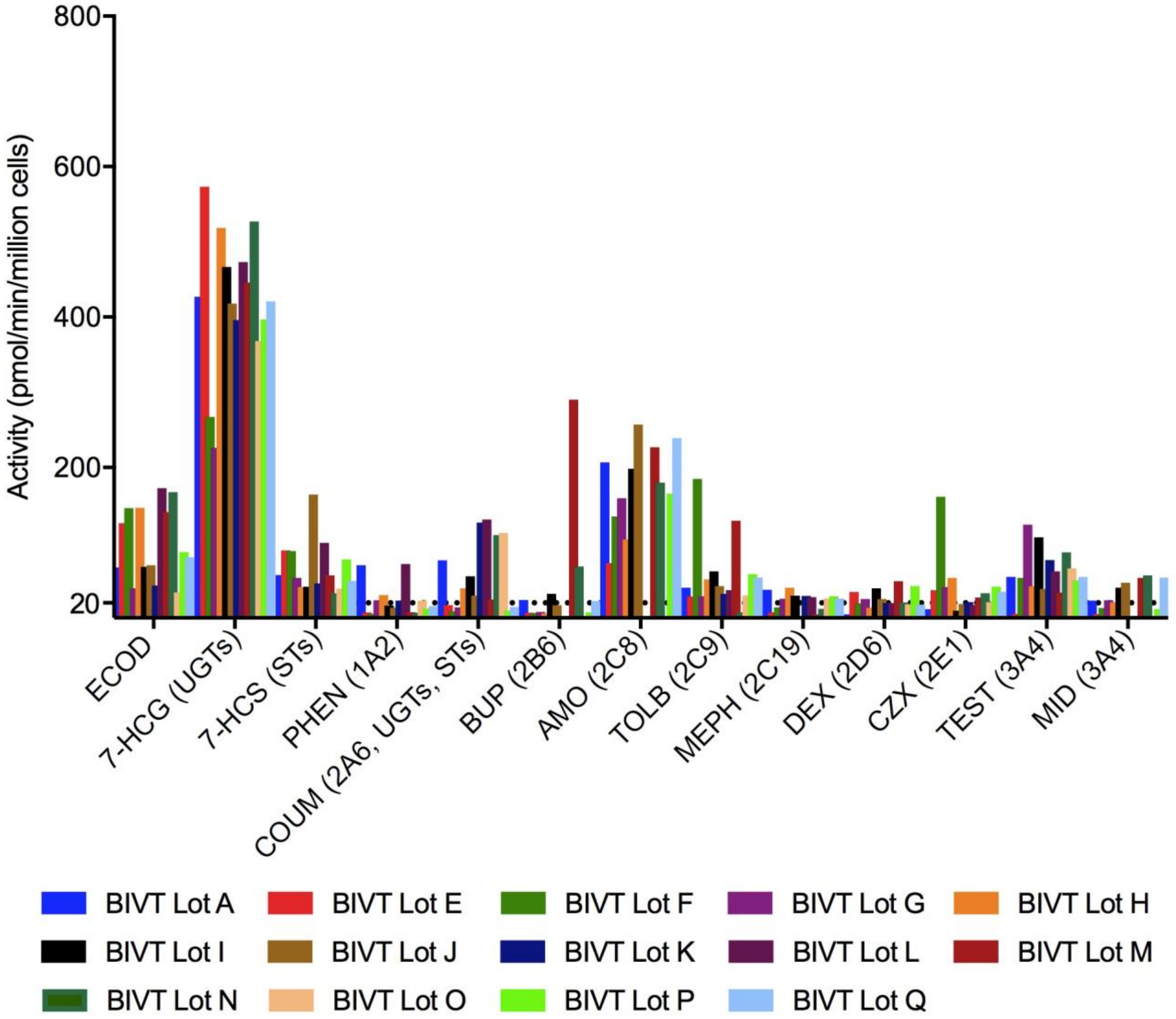

Supplementary Figure 5 | Enzymatic characterization of Bioreclamation IVT primary human hepatocytes (PHHs) donor lots. Basal metabolic activity of 14 BIVT donor lots was performed and provided by Bioreclamation IVT, Inc. using a validated panel. Donor lots show variation in metabolic activity. BIVT Lot A is PHH donor lot PDC.

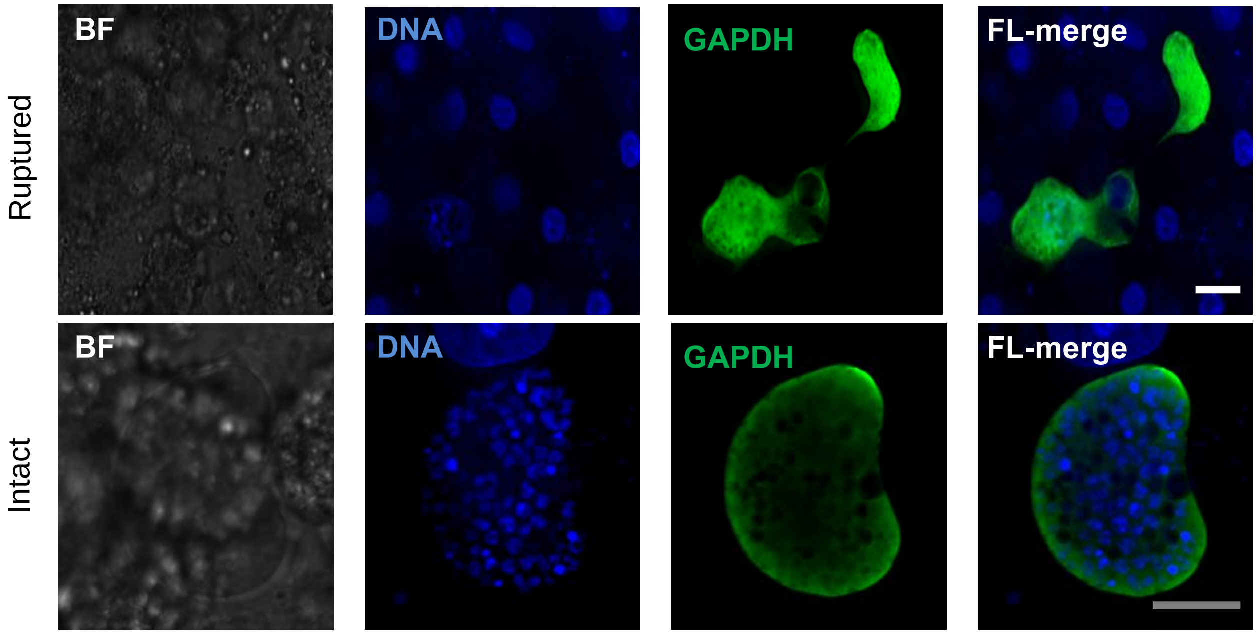

Supplementary Figure 6 | *P. falciparum* liver stage (LS) schizonts on day 8. LS schizonts begin to reach full maturation on day 7 with merozoite release beginning on day 7–8. However, not all schizonts mature at the same rate with some parasites continuing to develop. Scale bars, white, represents 5 µm and grey represents 10 µm.

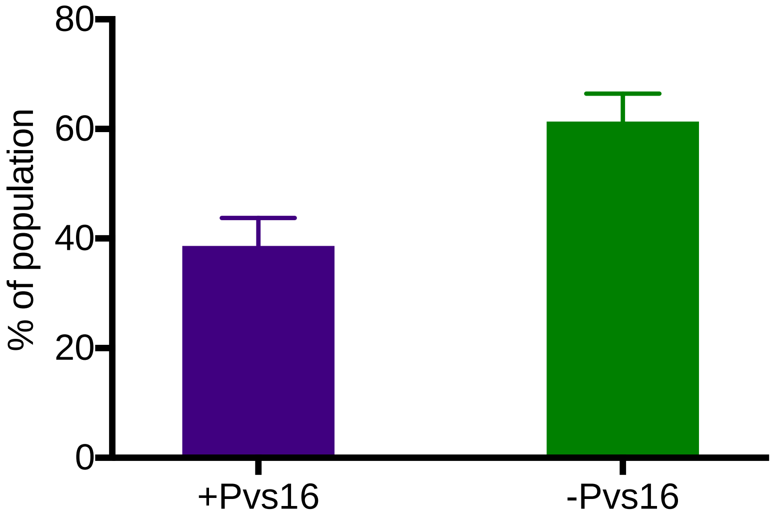

Supplementary Figure 7 | Day 8 *P. vivax* liver stage (LS) schizont immunofluorescent positive for anti-Pvs16. Quantification of immunofluorescent staining of day 8 *P. vivax* schizonts with anti-Pvs16 reveals unequivocal staining of schizonts. Graph bars represent means with s.d. from biological replicates (n = 2) and experimental replicates (n = 3).

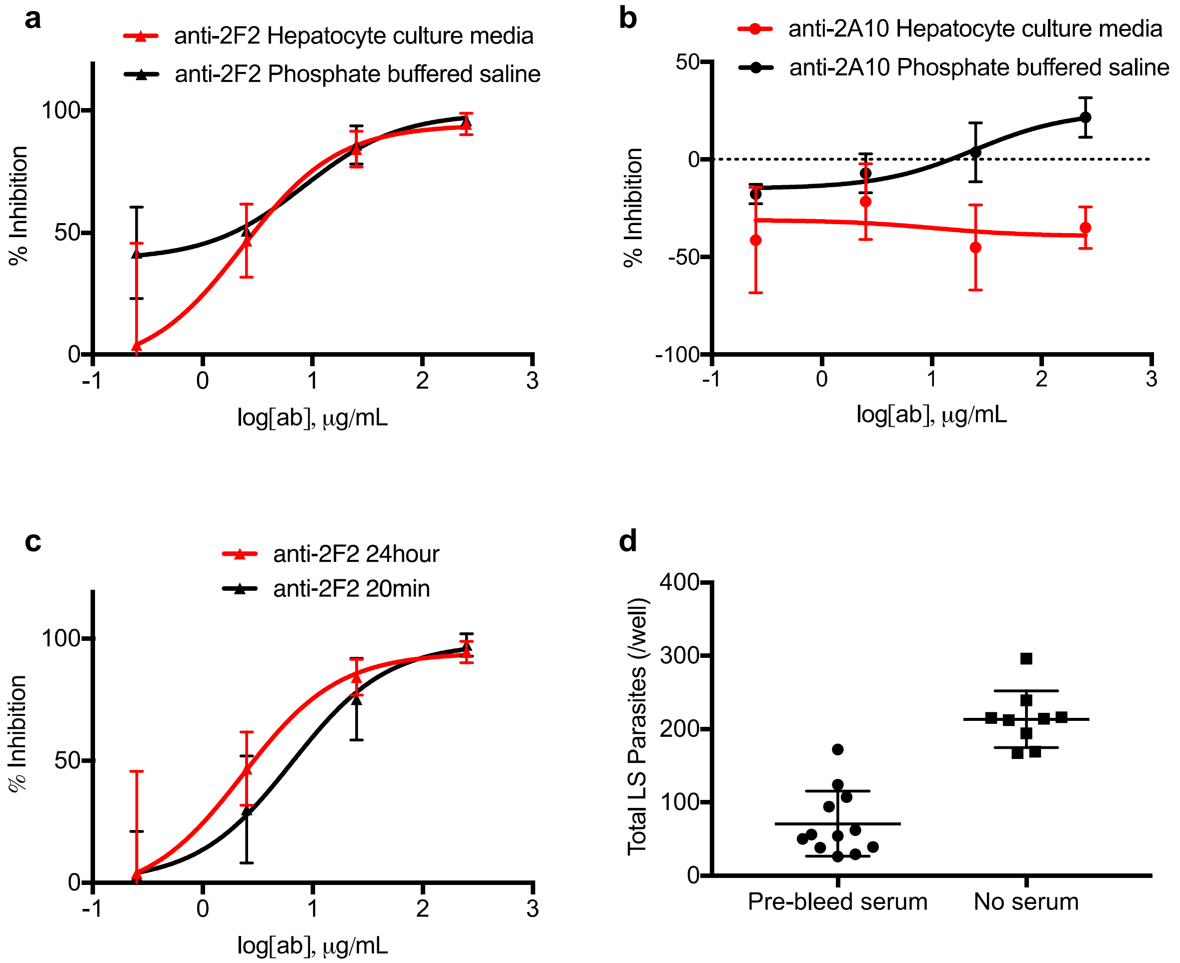

Supplementary Figure 8 | Media type effects *P. vivax* sporozoites viability, invasion, and LS development. **(a)** *P. vivax* sporozoites incubated in phosphate buffered saline (PBS) for 20-minutes at room temperature with anti-PvCSP mAB 2F2 showed an increased % inhibition compared to *P. vivax* sporozoites incubated in hepatocyte culture media (HCM), however, this likely unviable sporozoites leading to reduced invasion rates. **(b)** Similarly, *P. vivax* sporozoites incubated in phosphate buffered saline (PBS) for 20-minute at room temperature with a non-species-specific antibody (anti-PfCSP mAB 2A10) showed inhibition when there should not be an inhibitory effect. **(c)** Comparing 24 hours vs. 20 minutes, *P. vivax* sporozoites exposure to anti-PvCSP mAB 2F2 shows a slight curve shift, however, the data goodness of fit increases. **(d)** *P. vivax* sporozoites exposed to the control rabbit serum (pre-bleed) showed moderate inhibition in comparison to the no rabbit serum controls. Therefore, IC_{50} curves for screened sera samples (FMP014/ALF, FMP014/ALFQ, FMP014V/ALF, and FMP014V/ALFQ) were generated by accounting for existing inhibition from serum. Graph bars represent mean with s.d. from biological replicates (n = 3) and experimental replicates (n = 2).

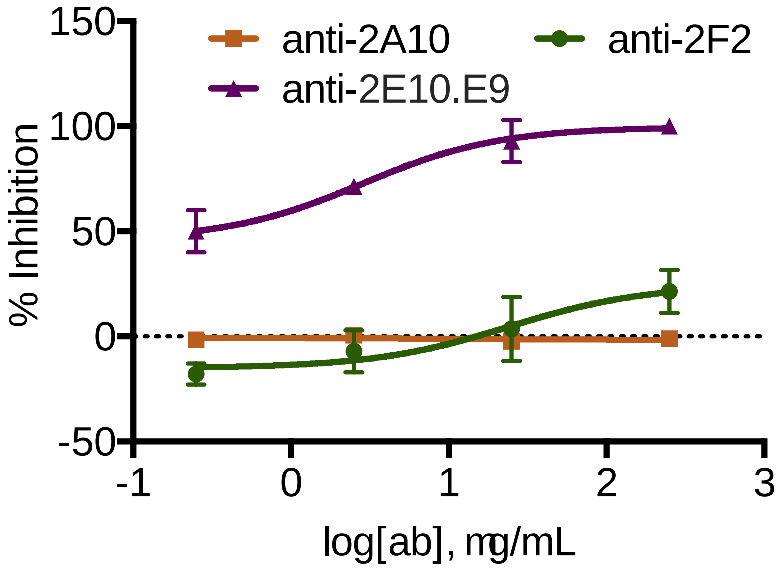

Supplementary Figure 9 | Use of cryopreserved *P. vivax* sporozoites **for ILSDAs**. *P. vivax* sporozoites exposed to anti-PvCSP mAB 2E10.E9 (VKS type 247) concentrations showed high inhibition (IC_{50} = 2.85 µg ml^{-1}) with no effect seen on *P. vivax* sporozoites exposed to anti-PvCSP mAB 2F2 (VKS type 210) or anti-PfCSP mAB 2A10 CSP, indicating cryopreserved sporozoites can successfully be used for ILSDAs. Graph bars represent means with s.d. from a biological replicate (n = 1) and experimental replicates (n = 2).

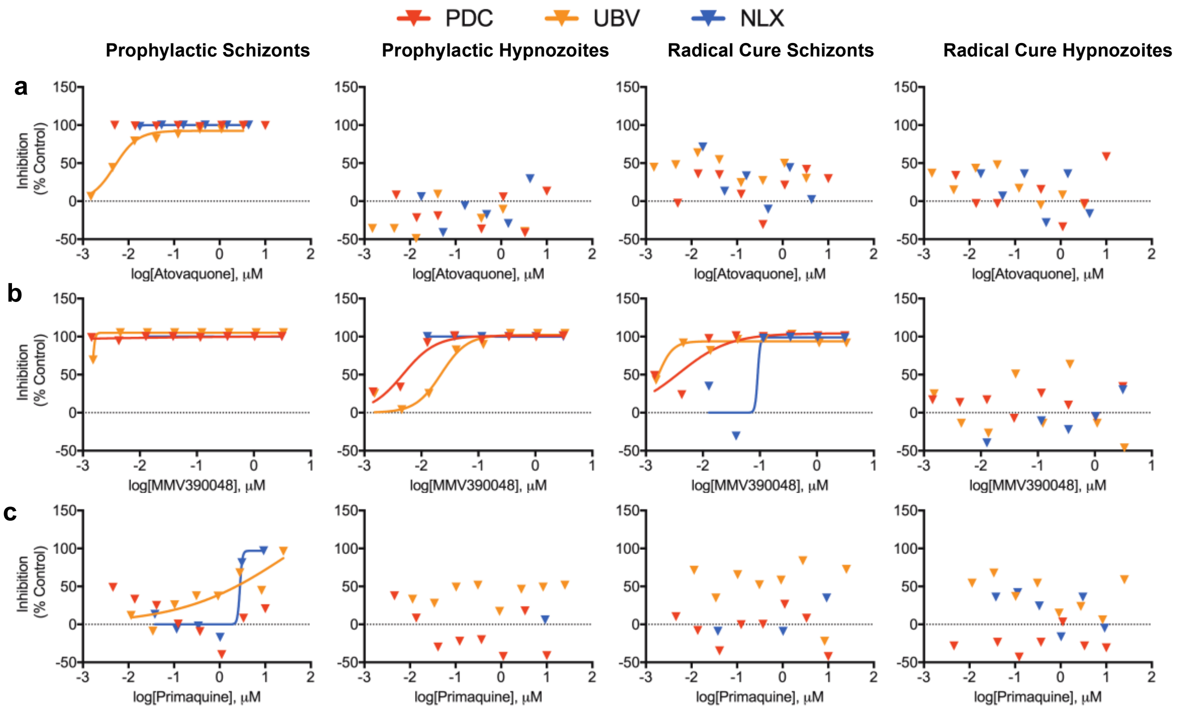

Supplementary Figure 10 | Comparison of *P. vivax* dose responses in three different primary human hepatocyte (PHH) donors. (**a)** Dose response charts after treatment with atovaquone in prophylactic and radical cure modes, in all donors atovaquone is only effective against young schizonts. (**b)** Dose response charts after treatment with PI4K inhibitor MMV390048 in prophylactic and radical cure modes, in all donors MMV390048 is active against all forms except mature hypnozoites. (**c**) Dose response charts after treatment with primaquine in prophylactic and radical cure modes. Primaquine is active against young schizonts in NLX and UBV PHH donor lots but not in PDC donor lot. Primaquine is metabolized by hepatocyte-resident CYP2D6, which is expressed at lower levels in PDC cells (metabolism of dextromethorphan into doxtrorphan, quantified by mass spectrometry: 1.75 pmol/min/10^6 cells) compared to NLX (14.4 pmol/min/10^6 cells) or UBV (25.2 pmol/min/10^6 cells). All data are of at least a biological replicate (n = 1) and experimental replicates (n = 2).

Supplementary Table 1 | Characterization of commercially available primary human hepatocyte (PHH) donor lots

PHH Source (company)	PHH donor	Viability[a]	Viability[b]	Total cells (#/vial) [a]	Total cells (#/vial) [b]	Supports *P. vivax* LS parasites	Notes
Bioreclamation IVT (BIVT)	PDC	84%	72%	4,700,000	5,000,000	+++	
	NLX	80%	95%	4,800,000	6,600,000	++	
	UBV	79%	94%		8,400,000	+++	
Company 2	Lot A	75%	53%	3,200,000	1,400,000	+	Fibroblasts after 2 weeks
	Lot B	80%	82%	2,600,000	2,600,000	+	Fibroblasts after 2 weeks
Company 3	Lot A	81%	76%	4,500,000	5,200,000	-	Fibroblasts by day 3 Hep lot used by CIDR in mice
	Lot B	55%	73%	6,300,000	5,600,000	+ (-)	Fibroblasts by day 3 Hep lot used by CIDR in mice
Company 4	Lot A	62%	53%	8,900,000	7,000,000	+	
	Lot B	68%	57%	10,700,000	8,400,000	+	
	Lot C		60%	4,900,000	6,100,000	+	Failed to seed as monolayer

[a, b] Denotes thaw replicates 1 and 2

+ Refers to IFA quantification of *P. vivax* LS parasites.

Supplementary Table 2. Enzymatic characterization of Bioreclamation IVT primary human hepatocytes (PHH) donor lots.

Substrate/ metabolite (CYP)	Abbrev.	BIVT Lot A	BIVT Lot E	BIVT Lot F	BIVT Lot G	BIVT Lot H	BIVT Lot I	BIVT Lot J	BIVT Lot K	BIVT Lot L	BIVT Lot M	BIVT Lot N	BIVT Lot O	BIVT Lot P	BIVT Lot Q
7-Ethoxycoumarin deethylation/7-Hydroxycoumarin (1A2, 2E1)	ECOD	67.3	126	146	39.5	146.5	68	69.9	43	172.7	140.7	167.4	34	87.4	80.5
7-Hydroxycoumarin/7-HC Glucuronide (2A6)	7-HCG (UGTs)	427	573	267.4	226.2	518.4	466.6	418.1	396	472.9	445.6	527.2	368	396.9	421
7-Hydroxycoumarin/7-HC Sulphate (2A6)	7-HCS (STs)	57.1	89.6	89	53.2	41.1	41.4	163.9	46	99.9	56.4	32.7	39	77.9	49.4
Phenacetin/Acetaminophen (1A2)	PHEN (1A2)	69.9	6.6	3.1	23.5	30.4	16.6	13.7	23	71.5	7.4	6.5	23	12.5	15.9
Coumarin/Total Metabolites (2A6)	COUM (2A6, UGTs, STs)	76.7	16.8	8.8	14.2	39.6	55.3	29.6	127	130.9	24.2	110.1	113	9.6	14.3
Bupropion/Hydroxybupropion (2B6)	BUP (2B6)	24	6.4	5.5	8	4.5	31.9	17.1	—	—	290.1	68.5	—	7.2	22.7
Amodiaquine/Desethylamodiaquine(2C8)	AMO (2C8)	207	73	135	159.1	104.2	198.1	257	—	—	226.9	180	—	165	239
Tolbutamide/4-Hydroxytolbutamide (2C9)	TOLB (2C9)	40.2	28.5	184.9	28.9	51.1	61.9	42.4	32	36.8	129.4	7.6	30	58.3	53.7
Mephenytoin/4-Hydroxymephenytoin (2C19)	MEPH (2C19)	37.5	7.2	13.9	25.4	40.3	29.7	0	29	27.4	1.8	11.9	26	28.8	26
Dextromethorphan/Dextrorphan (2D6)	DEX (2D6)	1.8	34.7	19.4	25.2	13.6	39.5	25.2	20	19.9	48.7	20.8	18	42.1	17.6
Chlorzoxazone/6-Hydroxychlozoxazone (2E1)	CZX (2E1)	11.5	36.9	161.3	40.9	53.3	9.6	18.5	21	16.9	27.3	32.7	21	41.3	34.9
Testosterone/6ß-Hydroxytestosterone (3A4)	TEST (3A4)	54.5	2.9	53.2	124	42.3	107.2	38.6	77	62	33.6	87	66	49.9	54.7
Midazolam/1-OH Midazolam (3A4)	MID (3A4)	23.2	2.7	13.3	23.5	20.6	40.4	46.7	—	—	53.3	56.6	—	11.8	53.5

Abbrev. means abbreviation of substrate. All data was performed and provided by Bioreclamation IVT Inc, (Baltimore, MD, U.S.A.).
— Denotes not applicable

Metabolic panel of 14 Bioreclamation IVT (BIVT) donor lots where BIVT Lot A is donor lot PDC.

Supplementary Table 3 | Functional characterization of Bioreclamation IVT cryopreserved primary human hepatocyte (PHH) donor lots.

PHH donor	Viability (thaw) [¥]	Total cells (#/vial)	Plated [†]	High TMRM mean intensity [a, b]	High TMRM mean area (μm^2) [a, b]	Low TMRM mean intensity [a, b]	Low TMRM mean area (μm^2) [a, b]	Active transport (%) [a, b]	Inactive transport (%) [a, b]	Total hepatocytes (#) [a, b]	Hepatocyte nuclei healthy (%) [a, b]	Hepatocyte nuclei unhealthy (%) [a, b]	Mean Pv LS (#/well) [±]	Pv LS mean schizont area (μm^2) [±]	Mean Pf LS (#/well) [±]	Pf LS mean schizont area (μm^2) [±]
PDC	84.60%	5,013,850	+	3,816, 3,313	196.45, 518.75	2,939, 2,812	0.31, 3.33	31.62, 42.72	68.39, 57.29	23,784, 12,817	93.06, 98.41	6.94, 1.59	20	800.73	30	101.53
HepRG	93.22%	6,676,500	+	3,472, 3,301	997.85, 1,736.50	2,635, 2,068	14.53, 270.20	25.40, 20.32	74.60, 79.68	22,584, 20,955	92.05, 88.96	7.95, 11.04	0	—	0	—
BIVTLotE	90.29%	7,479,800	+	3,682, 3,821	299.05, 218.10	2,930, 2,874	0.45, 2.16	28.34, 42.53	71.67, 57.47	20,527, 15,078	98.25, 99.29	1.75, 0.71	10	127.65	30	125.87
BIVTLotF	89.26%	5,940,000	+	3,503, 3,291	415.20, 1,938	2,856, 2,361	2.94, 74.58	18.04, 18.51	81.97, 81.49	17,073, 13,918	99.19, 96.72	0.81, 3.28	0*	*	0	—
BIVTLotG	77.06%	6,749,250	+	3,234, 3,269	281.50, 405.15	2,825, 2,160	3.68, 108.87	27.18, 28.85	72.83, 71.15	20,395, 13,801	98.71, 97.44	1.29, 2.56	20	1,721.13	0	—
BIVTLotH	79.24%	6,950,150	+	3,728, 3,334	210.10, 475.60	2,906, 2,764	3.61, 9.62	47.53, 50.05	52.48, 49.96	10,660, 4,152	87.91, 67.58	12.09, 32.42	0	—	100	78.56
BIVTLotI	77.63%	6,094,500	+	3,422, 3,201	228.45, 4,783	2,818, 2,586	6.76, 35.36	32.37, 36.79	67.63, 63.21	17,508, 13,037	94.68, 99.26	5.32, 0.74	0	—	20	151.54
BIVTLotJ	71.66%	6,534,000	+	3,881, 3,287	157.95, 1,158.85	2,939, 2,564	0.34, 64.78	37.48, 33.18	62.52, 66.83	20,976, 11,891	95.15, 99.39	4.85, 0.61	120	268.76	30	170.42
BIVTLotK	86.17%	4,608,900	+	3,529, 3,447	152.10, 1,490	2,906, 1,490	3.99, 311.30	54.99, 77.14,	45.01, 22.87	8,070, 3,740	96.85, 72.70	3.15, 27.30	40	3,163.04	10	149.78
BIVTLotL	71.13%	3,100,700	-	—	—	—	—	—	—	—	—	—	—	—	—	—
BIVTLotM	83.58%	8,988,000	-	—	—	—	—	—	—	—	—	—	—	—	—	—
BIVTLotN	90.32%	6,740,250	+	3,873, 3,404	1,436, 4,321	2,535, 1,730	26.75, 244.25	35.72, 33.93	64.28, 66.07	12,164, 9,357	95.31, 94.81	4.69, 5.19	—	—	0	—
BIVTLotO	73.30%	4,463,100	-	—	—	—	—	—	—	—	—	—	—	—	—	—
BIVTLotP	90.10%	7,550,400	+	3,885, 3,372	189.65, 271.05	2,943, 1,347	0.81, 200.90	49.96, 13.07	50.05, 52.27	17,130, 10,520	99.26, 98.30	0.74, 1.70	70	540.74	0	—
BIVTLotQ	81.52%	7,357,500	+	3,665, 3,529	1,099, 4,098	2,592, 1,694	21.85, 227.75	43.10, 47.74	56.90, 52.27	11,586, 5,963	94.56, 90.83	5.44, 9.16	160	2,244.85	—	—

¥ Determined at time of thaw with trypan blue exclusion count
† Refers to visual attachment of hepatocytes to well surface; + yes, — no
— Denotes not applicable
[a] Day 2 post seed, [b] Day 8 post seed
* Denotes high sporozoite invasion rate however failure of LS schizont development
± Determined by immunofluorescent staining and automated quantification or analysis on Operetta of experimental wells on Day 7 LS parasites

Screening of 13 additional Bioreclamation IVT (BIVT) donor lots revealed majority attach to the 384-well plate well but not all are susceptible to *Plasmodium* infection and development. However, donor lots with high *Plasmodium* infection rates appear to have a correlation with hepatocytes showing an increased area of TMRM activity (respiration) and active bile canaliculi transportation from day 2 to 8 post-seed. All 3 BIVT donor lots were assessed with the previously described live-imaging functional assays measuring bile canaliculi formation, growth, mitochondrial activity, and nuclei health for 14 days. The table shows day 2 and day 8 time points.

**Supplementary Table 4 | *Plasmodium* spp. liver stage (LS) parasite size distribution.**						
	P. falciparum		***P. vivax***			
**Size category**	**Mean parasite area [a] (μm²)**	**Population [a] (%)**	**Mean parasite area [a] (μm²)**	**Population [a] (%)**	**Mean parasite area [b] (μm²)**	**Population [b] (%)**
1	70.90	75.96	32.87	65.51	36.22	60.98
2	133.13	22.13	198.45	6.78	160.53	2.41
3	310.30	0.27	403.37	7.59	397.65	1.07
4	-	-	709.14	16.32	748.18	3.10
5	-	-	1259.10	3.78	-	-
6	-	-	-	-	2706.96	32.44

[a] Denotes day 6 LS parasite
[b] Denotes day 8 LS parasite

Plasmodium spp. mean parasite size distribution was calculated from biological replicates (n = 3) with experimental replicates (n = 26, *P. vivax*) or (n = 8, *P. falciparum*). Size category was assigned by HCI thresholds defined previously (Table 1) where day 6 *P. falciparum* does not exceed size category 3.

Supplementary Table 5 | Calculated EC_{50} for MMV compounds targeting *Plasmodium* liver stage (LS) parasites

Compound ID	Compound name	*P. falciparum*[a]	*P. vivax* Schizonts[a]	*P. vivax* Hypnozoites[a]	*P. vivax* Schizonts[b]	*P. vivax* Hypnozoites[b]
		EC_{50}*	EC_{50}*	EC_{50}*	EC_{50}*	EC_{50}*
MMV000147	P218	< 0.012	0.045	> 10	> 10	> 10
MMV669059	DSM421	0.31	0.021	> 10	> 10	> 10
MMV675887	AN13762	0.041	3.25	> 10	> 10	> 10
MMV6747751	KAF156	0.41	0.0168	0.18	0.014	> 10
MMV000024	Pyrimethamine	0.026	> 10	> 10	> 10	> 10
MMMV00046	Atovaquone	< 0.005	< 0.005	> 10	> 10	> 10
MMV390048	MMV048	0.011	< 0.005	< 0.039	0.012	> 3.3

*Denotes a concentration of µM
[a] Denotes prophylactic mode
[b] Denotes radical cure mode

P. falciparum and *P. vivax* LS parasites were tested in prophylactic mode, meaning drug treatment was initiated 24 hours after sporozoite invasion with continued drug until day 3 post infection and final fixation on day 6. EC_{50} values were calculated from biological replicates (n = 2, *P. falciparum* or n = 3, *P. vivax*) with experimental replicates (n = 1).

Supplementary Table 6 | Calculated EC_{50} for select ionophores targeting *P. vivax* liver stage (LS) parasites

	**Schizont**	**Hypnozoite**
**Compound name**	**EC_{50}***	**EC_{50}***
Monensin	0.013	0.007
Nigericin	< 0.005	< 0.005
Salinomycin	0.026	< 0.005
Lasalocid-A	0.095	0.064
Primaquine	2.695	> 10

* Denotes a concentration in µM

P. vivax LS parasites were tested in prophylactic mode with exposure to ionophores, each showing high efficacy against all LS parasite forms.
